# Factors determining chromosomal localization of transposable elements in plants

**DOI:** 10.1111/plb.70057

**Published:** 2025-05-29

**Authors:** E. Kejnovsky, P. Jedlicka, M. Lexa, Z. Kubat

**Affiliations:** ^1^ Department of Plant Developmental Genetics Institute of Biophysics of the Czech Academy of Sciences Brno Czech Republic; ^2^ Faculty of Informatics Masaryk University Brno Czech Republic

**Keywords:** Centromere, chromosomes, plant genome, recombination, transcription factor, transposable elements

## Abstract

Transposable elements (TEs) constitute a significant part of plant genomes and shape their genomic landscape. While some TEs are ubiquitously dispersed, other elements specifically occupy discrete genomic loci. The evolutionary forces behind the chromosomal localization of TEs are poorly understood. Therefore, we first review specific chromosomal niches where TEs are often localized including (i) centromeres, (ii) (sub)telomeres, (iii) genes, and (iv) sex chromosomes. In the second part of this review, we focus on the processes standing behind non‐equal distribution of various TEs in genomes including (i) purifying selection, (ii) insertion site preference or targeting of TEs, (iii) post‐insertion ectopic recombination between TEs, and (iv) spatiotemporal regulation of TE jumping. Using the combination of the above processes, we explain the distribution of TEs on sex chromosomes. We also describe the phenomena of mutual nesting of TEs, epigenetic mark silencing in TEs, and TE interactions in the 3D interphase nucleus concerning TE localization. We summarize the functional consequences of TE distribution and relate them to cell functioning and genome evolution.

## Graphical abstract

We review transposon chromosomal niches, and the functional consequences of their specific chromosomal localization in plants.

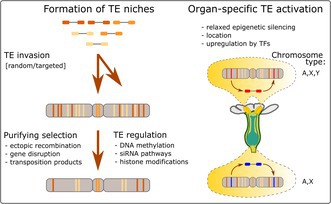

## INTRODUCTION

A significant part of eukaryotic genomes is made up of transposable elements (TEs), where in animals they can constitute more than 50% of the genome (e.g. non‐LTR TEs in human or grasshopper) (Canapa *et al*. 2015; Li *et al*. 2024), while in plants TEs constitute up to 90% of genomes in some species (Charles *et al*. 2008; Schnable *et al*. 2009; Wicker *et al*. 2018; Liehr 2021). However, the repeat content in plant genomes is quite variable and ranges from 9% in *Genlisea aurea* to 95% in *Pinus sylvestris* (Novak *et al*. 2020). Most comparative studies find a positive correlation between genome size and the proportion of TEs (Elliott & Gregory 2015; Macas *et al*. 2015), but this is only true for small and medium‐sized genomes. An explanation is offered by a systematic study of repetitive content in 101 plant species by Novak *et al*. (2020), which shows that in large genomes above approximately 10 Gbp, the proportion of repeats decreases to about 55% while the proportion of unique unclassifiable sequences increases. The authors explain this by epigenetic regulation (heterochromatinization) of (retro)transposons leading to a reduced rate of recombination‐based removal of repeats, which itself is a characteristic of species with giant genomes (Tiley & Burleigh 2015). Repeats trapped in regions of low recombination therefore degenerate and slowly transform into repeat‐derived unique DNA, ultimately unrecognizable as such. This consequently leads to an underestimation of the true proportion of DNA that is derived from repeats.

In eukaryotic genomes, transposons with a replicative mode of transposition dominate. These include Class 1 TEs, i.e. retrotransposons, as well as some representatives of Class 2, especially Helitrons. In plant genomes, Class 1 LTR retrotransposons tend to be the most abundant, with a 7–70% share and variable proportion of Ty3/gypsy to Ty1/copia superfamilies (Kejnovsky *et al*. 2012; Puterova *et al*. 2018; Wicker *et al*. 2018; Li *et al*. 2019; Liu *et al*. 2020; Quesneville 2020; Jesionek *et al*. 2021; Cai *et al*. 2022). The proportion of LTR retrotransposon (super)families is usually skewed by one or a few clades of recently spreading high copy number elements in each species. For example, closely related species of the genus *Phaseolus* contain markedly different ratios of Ty3/gypsy (2%–23%) to Ty1/copia (1%–7%) superfamilies, with variability skewed heavily mainly by Ty3/gypsy *Ogre* family elements (Ribeiro *et al*. 2020). Unlike in animals, non‐LTR retrotransposons only rarely occupy more than 5% of plant genomic space with some exceptions (Casacuberta & Santiago 2003). Class 2 DNA transposons with cut‐and‐paste mobilization mechanisms usually occupy only 1%–10% of the genome, but are relatively abundant in some species. For instance, the bread wheat genome contains about 16% *CACTA* DNA transposons, the same proportion as all Ty1/copia elements (Wicker *et al*. 2018). Helitrons, Class 2 elements with a rolling circle duplicative transposition, are another example of TEs with highly variable abundance. While being detected in a few copies in many plant genomes, they constitute over 6% of nuclear DNA in *Arabidopsis thaliana* and even 14% in several *Brassica rapa* cultivars (Quesneville 2020; Cai *et al*. 2022). The considerable variation in the ratios of individual TEs in closely related species indicates a continued high rate of insertion activity of TEs (Cegan *et al*. 2012). Perhaps even more indicative, however, are intraspecific differences in the abundance of TEs, such as DNA transposons in rice (Roffler & Wicker 2015) or LTR retrotransposons in the dioecious plant *Silene latifolia* (Puterova *et al*. 2018).

TEs often colonize specific genomic niches and interact structurally and functionally with other genomic components like DNA satellites, rDNA loci or genes. Due to their interactions with genes, TEs are often embedded in cellular regulatory networks (Feschotte 2008). The interactions of TEs with other repetitive elements play a role in 3D organization of the interphase nucleus and have recently been a subject of intensive study (Lu *et al*. 2021). The participation of various processes determining the localization of TEs and the factors playing a role in the formation of the genomic landscape are not well understood.

In this review, we summarize how plant transposable elements participate in (i) the constitution of a functional centromere, (ii) contribute to the chromosome end protection, (iii) are associated with genes, and (iv) accumulate in non‐recombining regions of the genome, such as the Y chromosome. We also describe the processes underlying the distribution of these repeats including (i) purifying selection, (ii) TE targeting and nesting, (iii) ectopic recombination, and (iv) sex‐specific transposition. Finally, we describe the functional consequences of TE distribution. Suppose the reviewed phenomenon is not yet known in plants. In that case, we discuss the situation in other organisms (e.g. telomeric retrotransposons in animals) to get a complex picture of localization of TEs in all chromosomal loci.

## CHROMOSOMAL LOCI OF TRANSPOSABLE ELEMENT ACCUMULATION

### Centromeric LTR retrotransposons

The centromeric and pericentromeric regions of plant monocentric and holocentric chromosomes are colonized by various LTR retrotransposon families belonging to either Ty3/Gypsy or Ty1/Copia superfamilies (summarized in Naish & Henderson 2024), where CRM (centromeric retrotransposon in maize) is the dominating Ty3/Gypsy family (Neumann *et al*. 2011, Fig. 1a). The CRM family can be divided into three clades (A, B and C) based on differences in the integrase DNA sequence and the presence of a putative targeting domain (PTD). These mutually related retrotransposons show both centromeric and ubiquitous chromosomal distribution. Namely, the members of group A possess the CR motif in their integrase sequence and are concentrated in the centromeric region, while members of group B and C are dispersed throughout the genome. Chromodomain of integrase is crucial for targeting heterochromatin since it recognizes histone H3K9 methylation, an epigenetic mark characteristic of heterochromatin (Gao *et al*. 2008). The CRM elements represent an active component of centromeres in a wide range of angiosperm species and play a role in centromere evolution (Neumann *et al*. 2011).

**Fig. 1:**
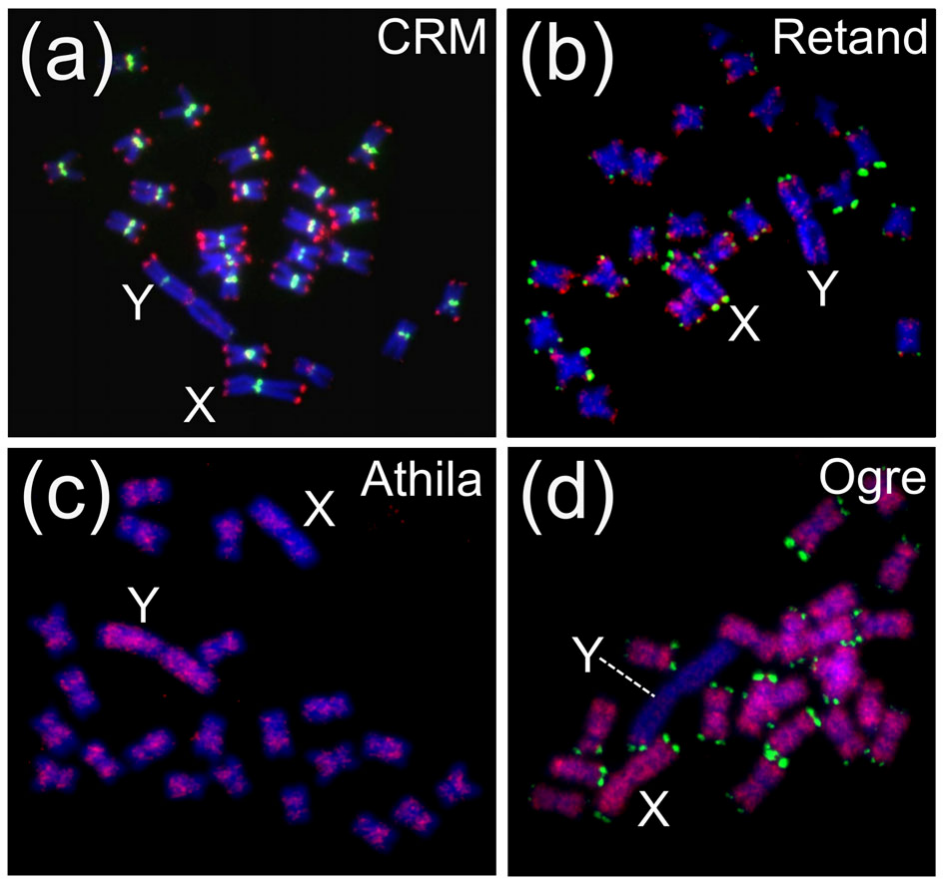
Contrasting patterns of transposable elements chromosomal localization by FISH in dioecious model plant *Silene latifolia*, possessing XY chromosomes. (a) centromeric localization of CRM centromeric LTR retrotransposon (green), (b) subtelomeric localization of Retand LTR retrotransposon (red), (c) accumulation of Athila retrotransposon (red) on the Y chromosome, (d) absence of Ogre LTR retrotransposon (red) on the non‐recombining Y chromosome, Subtelomeric satellite X43.1 is in green (b,d) or red (a).

Furthermore, centromeres can be invaded by other Ty1/Copia or Ty3/Gypsy retrotransposon families. For example, Ale elements (belonging to Ty1/Copia retrotransposons) are present in (peri)centromeres of *Brassica rapa* along with large satellite arrays and CRM elements (Zhang *et al*. 2023). Moreover, there are differences in invasion times of TEs into centromeres and pericentromeres in *Brassica rapa*, revealing the diverse colonization mechanisms of these two neighbouring compartments (Zhang *et al*. 2023). Centromeres contain the youngest insertions (0.14 MYA avg), presumably due to rapid turnover, i.e. the incorporation of new copies leading to the degradation of older ones. Pericentromeres, on the contrary, contain the oldest insertions (0.51 MYA avg) compared to the whole‐genome average of 0.32 MYA. This may be due to the low rate of removal via ectopic recombination that is characteristic for pericentromeres, or the rapid amplification of LTR retrotransposons in the centromeres could force the old LTRs out, leading to the residence of relatively old LTRs in the flanking pericentromeres (Zhang *et al*. 2023).

In some extreme cases, the centromeric regions are occupied only by retrotransposons, as shown recently in domesticated einkorn, *Triticum monococcum* (Ahmed *et al*. 2023; Heuberger *et al*. 2024). The centromere‐associated autonomous retrotransposon family *RLG_Cereba* in the functional centromeres mobilizes the non‐autonomous *RLG_Quinta* elements. Outside the functional centromeres, these retrotransposon families are rare and other TE families dominate (Ahmed *et al*. 2023). In *Arabidopsis thaliana*, the centromeric satellite arrays are invaded by specific Ty3/Gypsy *Athila* clades (Wlodzimierz *et al*. 2023). The authors proposed that *Athila* integrases can recognize centromeric DNA and/or its chromatin state. Furthermore in *Arabidopsis*, the other members of LTR and non‐LTR retrotransposons tend to cluster with pericentromeric heterochromatin of all chromosomes that is a result of (i) selection against integration into euchromatin, and (ii) accumulation of elements due to suppression of recombination (Peterson‐Burch *et al*. 2004).

The active role of CRM retrotransposons in functional centromeres and the process of cell division, as well as the intermingling of retrotransposons with satellites in centromeres, is supported by the finding of completely different organization of these repeats in genomes of holocentric species (Hofstatter *et al*. 2022). The retrotransposons together with satellites are more or less evenly distributed along the entire length of chromosomes in holocentric species in contrast to their discrete localization in functional centromeres in monocentrics (Hofstatter *et al*. 2022).

The debates about the role of retrotransposon insertions into centromeres or pericentromeres are ongoing. It is unclear whether retrotransposons target centromeres because these regions provide a “safe” genomic environment or whether these elements actually play an active role in centromere function (Birchler & Presting 2012). In fact, a scenario in which both possibilities are valid at the same time may be possible. In *A. thaliana*, a specific clade of Athila retrotransposons invaded the centromere of chromosome 5 recently. Athila elements give rise to short interfering RNAs (siRNAs) that direct DNA methylation of other genomic copies of Athila retrotransposons. Shimada *et al*. (2024) propose that insertion of Athila elements that are targets of these siRNAs results in silencing of centromeric transcription. In turn, the silencing is required for correct condensation of pericentromeres and proper mitotic chromosome segregation. The centromere thus becomes dependent on the presence of Athila elements and represents a safe space for their incorporation. Similar strategies may underlie the localization of TEs in maize (Gent *et al*. 2017) and yeast (Niki 2014) whose centromeres have also been recently invaded by retrotransposons. The repeated TE invasion into centromeres observed in eukaryotes may reflect general effects of TEs on the DNA locus in which they are located. Thus, the occurrence of TEs in non‐recombining heterochromatic regions of the genome is not a consequence, as previously suggested, but rather TEs themselves are the cause of heterochromatinization and reduced recombination frequency (reviewed by Kent *et al*. 2017; Choi & Lee 2020). A growing number of studies in both plants and animals favour this notion. For example, loci with retrotransposon insertions show reduced meiotic recombination frequency compared to homologous loci without insertions in Drosophila (Huang *et al*. 2024), and a similar suppressive effect of retrotransposons on recombination has been demonstrated in regions both inside and outside the centromeric and pericentromeric regions in tomato (Fuentes *et al*. 2022) and Arabidopsis (Underwood *et al*. 2018; Fernandes *et al*. 2024). The suppressive effect is associated mainly with retrotransposons, which unlike DNA transposons, are targets of intensive siRNA‐mediated silencing. Conflicting reports about TE effects on chromatin state are likely caused by variable levels of epigenetic silencing regulating various TE clades with different copy numbers and replicative mechanisms as reviewed by Underwood & Choi (2019). Overall, retrotransposons may accumulate in regions of the genome where heterochromatinization and reduced recombination frequency are preferred by the host itself. Retrotransposons also help the host to maintain its preferred state in these regions or help compensate for genetic mutations that disrupt it (Shimada *et al*. 2024). This idea is consistent with pericentromeric regions of species with large chromosomes showing low meiotic recombination rates that increase towards subtelomeres with higher gene density. This gradient is not observed in species with small chromosomes such as Arabidopsis (Haenel *et al*. 2018; Dukić & Bomblies 2022; Schreiber *et al*. 2022). Therefore, at least in some species, (peri)centromeres can be considered a safe region with a low probability of TE removal, consistent with their higher density and age of insertions there. Moreover, insertions into (peri)centromeres reduce the likelihood of causing lethal knockout mutations in their host. From the perspective of the retrotransposon, such insertions prevent recombination‐mediated loss of retrotransposons.

### Telomeric and subtelomeric retrotransposons

Telomeres represent another remarkable functional part of the eukaryotic genome. They protect chromosome ends against shortening during replication. While telomere‐associated transposable elements are common in animals, protists and fungi, they are rare in plant species, as exemplified by Penelope‐like elements in spike moss, *Selaginella moellendorffii* (Gladyshev & Arkhipova 2007), or subtelomeric Retand LTR retrotransposons in white campion, *Silene latifolia* (Kejnovsky *et al*. 2006).

Telomeric associations have been identified in the special group of Penelope‐like elements (PLE) in two bdelloid species, *Adineta vaga* and *Philodina roseola* (Gladyshev & Arkhipova 2007). *PLE*s are a superfamily of eukaryotic retroelements characterized by a reverse transcriptase domain with similarities to telomerases and group II introns. These elements constitute a novel superfamily of eukaryotic retroelements, different from non‐LTR and LTR retrotransposons. PLEs were found in diverse eukaryotes including bdelloid rotifers, basidiomycete fungi, amoebae, flatworms, roundworms, fish, amphibia, reptilia and plants (Evgen'ev & Arkhipova 2005; Arkhipova 2006). PLEs insert relatively randomly throughout the genome, preferring AT‐rich targets.

In Drosophila and some other animal species, non‐canonical telomeres formed by several types of non‐LTR retrotransposons were found. These telomeres were composed solely by three retrotransposons—Het‐A, TAHRE and TART forming HTT arrays at chromosome ends (Mason *et al*. 2008). Similarly to common telomeric tandem repeats containing runs of guanine and forming DNA quadruplexes, these telomeric retrotransposons also contain strand‐asymmetric runs of guanines to fulfil the same telomere protection role (Jedlička *et al*. 2023). The presence of G4‐forming motifs is probably one of the prerequisites for the recruitment of specific retrotransposons to chromosome ends. In plants, no similar telomeric retrotransposons have been found so far.

Related transposable elements residing in telomeres are called Terminons (Arkhipova *et al*. 2017). These giant elements can attach to G‐rich telomeric repeat overhangs at the chromosome ends, in a process apparently facilitated by complementary C‐rich repeats at the 3′‐end of the RNA template immediately adjacent to a hammerhead ribozyme motif. Terminon unit, which can exceed 40 kb in length, is an unusually complex and diverse structure, and can form very long chains, with host genes often captured between units. Terminons contain Athena reverse transcriptases previously described in bdelloid rotifers and belonging to the enigmatic group of Penelope‐like elements (Arkhipova *et al*. 2017).

The general pattern of subtelomeric composition in plants could be inferred from a recent detailed study on wheat (Aguilar & Prieto 2020). The subtelomeric regions revealed chromosome‐specific distribution patterns of diverse LTR retrotransposons (Ty1/Copia, Ty3/Gypsy and DIRS1), DNA transposons, genes and satellite repeats. The terminal regions of chromosomes, which include telomeres and subtelomeres, participate in chromosome recognition and pairing during meiosis in this species (Aguilar & Prieto 2020).

**Fig. 2:**
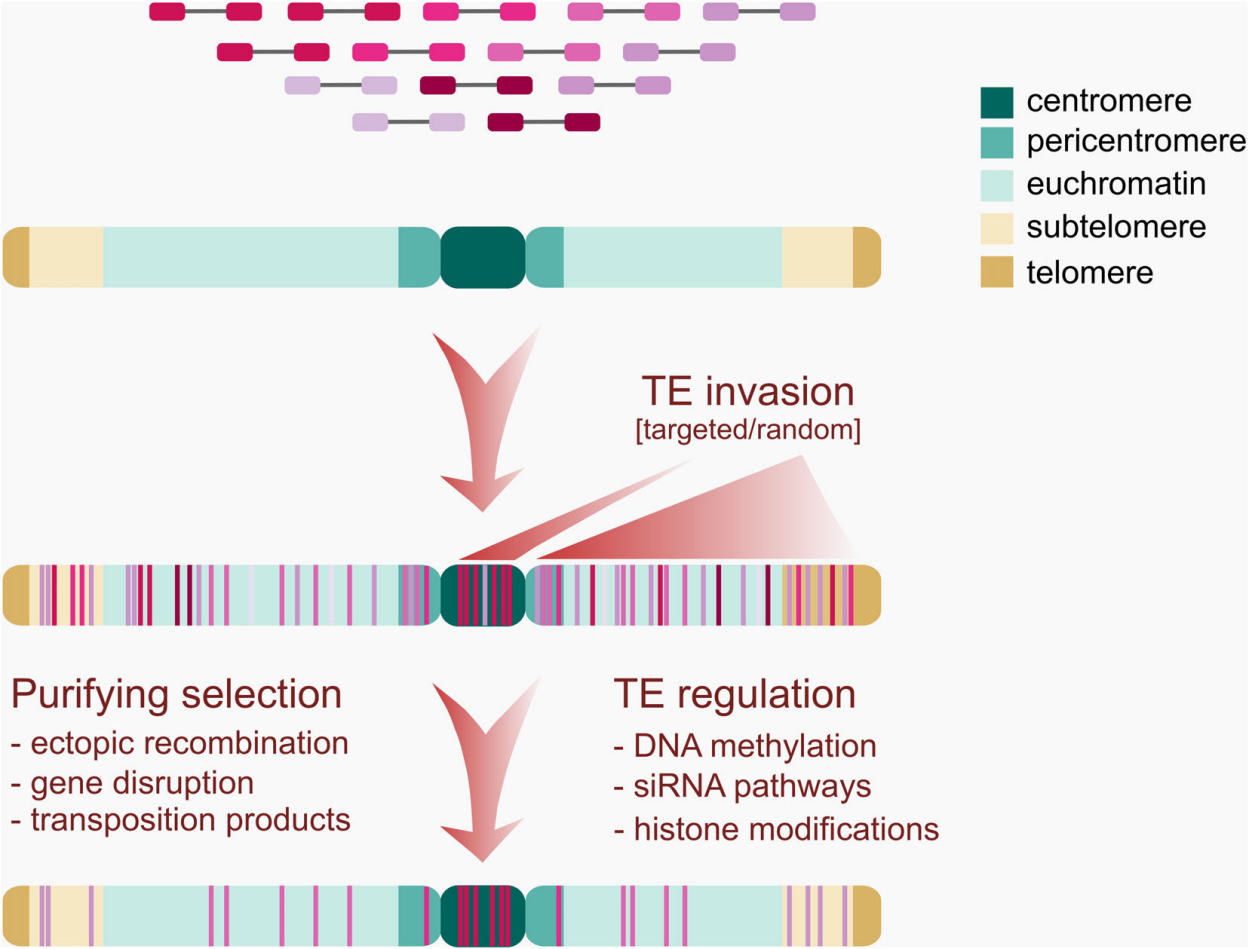
Reasons of transposable elements gathering in specific chromosomal niches. TEs are either randomly inserted along the whole chromosome but are later retained only in specific niches, or TEs are targeted into centromeres and therefore are localized only in these chromosomal niches.

Interestingly, in a dioecious plant white campion (*Silene latifolia*) a subtelomere‐specific retrotransposon Retand (Ty3/Gypsy superfamily) has been identified (Kejnovsky *et al*. 2006). Retand occupies subtelomeres of all autosomes, the X chromosome and one subtelomere of the Y chromosome (Figs. 1b and 2). The Retand family consists of autonomous elements with *gag* and *pol* genes as well as non‐autonomous elements having only an LTR with expanded STR1 tandem repeats between them (Kejnovsky *et al*. 2006). These elements demonstrate that satellites can be spread in the genome by transposable elements and 3′UTR of plant LTR retrotransposons can serve as a source of novel satellite repeats (Macas *et al*. 2009). This scenario is confirmed by a more recent analysis showing that 9 of the 11 major satellites originated from short tandem repeats within the LTR retrotransposons of *Lathyrus sativus* (Vondrak *et al*. 2020).

## ASSOCIATION OF TRANSPOSABLE ELEMENTS WITH GENES

MITEs (miniature inverted repeat transposable elements) represent a unique group of transposable elements that are non‐autonomous partners of DNA transposons of the Tc1/Mariner family (Bureau & Wessler 1992). They have a remarkable feature of colocalization with genes. MITEs are mostly found within introns of genes or in the gene vicinity, indicating a potential role of these elements in gene regulation (Wessler 1995). MITEs can be distinguished from other transposable elements by their small size (200–500 bp) and short terminal inverted repeats (TIRs) that are bound by transposase. Although MITEs were first discovered in plants, they have later been isolated from a wide range of eukaryotic organisms. MITEs can be divided into *Tourist*‐like, *Stowaway*‐like, and *pogo*‐like groups, according to similarities of their TIRs and TSDs (target site duplications). For instance, the *Tourist* element is known only from grasses, the *Emigrant* family only from *Arabidopsis*, and *Bigfoot* only from *Medicago* (Bureau & Wessler 1992, 1994; Casacuberta *et al*. 1998; Charrier 1999). Interestingly, mPing MITE element discovered by analysis of rice genome and belonging to the *Tourist‐like* group, is also active in *Arabidopsis thaliana* and inserts into genes or near genes (Yang *et al*. 2007). Moreover, MITE inserted in 3′‐UTR of rice Ghd2 gene (engaged in agronomic variables such as grain quantity and plant height), has been proven to repress its translation (Shen *et al*. 2017).

The high sequence identity observed for many MITE families indicates that these families might have spread recently throughout their respective host genomes. Also, the very high copy numbers attributed to many MITE families may be a result of independent amplifications of different subfamilies in the same genome (Feschotte *et al*. 2007). Despite MITEs being very common in plant genomes, there is little available literature on their origin and transposition mechanism. For instance, the high similarity between the inverted repeats of the Emigrant MITE in *Arabidopsis* and those of the Wujin in the yellow fever mosquito suggests the possibility of vertical transmission, indicating an ancient association of MITEs with eukaryotic genomes. However, Casacuberta *et al*. (1998) ruled out this possibility while suggesting that the similarity of terminal inverted repeats (TIRs) may also result from convergent evolution driven by constraints imposed by their interactions with conserved cellular machinery. Besides, the P instability factor (PIF), an active DNA transposons from maize together with a related miniature PIF (mPIF) with the same TIRs are probably responsible for the origin and spread of Tourist‐like MITEs (Zhang *et al*. 2001).

Non‐LTR retrotransposons are another TE group often found near host genes. In contrast to LTR retrotransposons, non‐LTR retrotransposons are relatively scarce in plants (Zhao *et al*. 2016), usually accounting for less than 5% of the genome with some exceptions, e.g., apple (*Malus domestica*, 7.95%, Velasco *et al*. 2010), sacred lotus (*Nelumbo nucifera*, 6.4%, Ming *et al*. 2013), sugar beet (*Beta vulgaris*, 5.67%, Dohm *et al*. 2014), and banana (*Musa acuminata*, 5.41%, D'Hont *et al*. 2012). Non‐LTR retrotransposons are frequently inserted in the upstream region of MYB transcription factors in plants where they directly influence the sub−/neo‐functionalization of these transcription factors (Zhang *et al*. 2023). Non‐LTR retrotransposons also play a role in the activation of anthocyanin biosynthesis via MYB transcription factors in *Capsicum annuum* (Jung *et al*. 2019).

Another group of DNA transposons with a specific chromosomal distribution is represented by Helitrons, a class of transposable elements initially discovered in the *Arabidopsis thaliana* genome and later found in all studied plant and many animal species (Kapitonov & Jurka 2001; Yang & Bennetzen 2009a). Helitrons are characterized by a 5′ TC terminus and 3′ CTRR terminus accompanied by a small hairpin near the 3′ end. These elements encode Rep/helicase‐like and RPA‐like (Replication protein A) proteins that are involved in the transposition process, probably using the rolling circle mechanism. The striking feature of plant Helitrons is their ability to capture gene fragments (Morgante *et al*. 2005) caused by malfunction or deletion of the 3′ termination signal, which allows polymerase reading beyond the original terminus (Barro‐Trastoy & Köhler 2024). Sixty percent of maize Helitrons have captured fragments of nuclear genes. Gene fragment acquisition appears to positively influence element survival and its ability to acquire additional gene fragments, however, Helitrons with gene fragments in the antisense orientation have a lesser chance to survive. Moreover, Helitrons preferentially insert near other Helitrons (Yang & Bennetzen 2009b). By combining gene fragments, these elements can generate genomic changes resulting in developmental novelties (Barro‐Trastoy & Köhler 2024). Helitrons affect plant stress responses as exemplified by the nonautonomous Helitron ATREP2 from Arabidopsis that is enriched for sequences from genes regulating the induced resistance response to herbivores.

There is also functional cross‐talk between TEs and genes. For example, in Arabidopsis, many genes are regulated through partial and fortuitous complementarity to siRNAs generated from TEs. The siRNAs generated by the Athila LTR retrotransposon target many genes (McCue *et al*. 2013). These siRNAs may suppress the expression of normal genes but enhance the expression of TEs. Thus there is a trade‐off between defence against TE activity and host gene expression, where TEs have been utilizing the silencing system for their survival in the resulting arms race.

## SEX CHROMOSOMES—NICHES OF TE ACCUMULATION OR ABSENCE

A special distribution of LTR retrotransposons is observed on the sex chromosomes of dioecious plants. According to the classical theory of sex chromosome evolution, TEs accumulate on the Y chromosome because they cannot be continuously removed due to the reduced recombination rate (Charlesworth 1991). However, cytological studies have revealed a surprising complexity: TEs not only accumulate on the Y but some clades (i.e. specific clades within Athila subfamily of LTR retrotransposons) are also almost absent on the Y chromosome in certain species. In addition, TE clades accumulated on the X chromosome have also been found. It should be noted, however, that these chromosomal distributions have so far only been detected in a few dioecious species with large and evolutionary young sex chromosomes, such as *Silene latifolia* possessing the XY chromosomes (Fig. 1c, Cermak *et al*. 2008; Filatov *et al*. 2009; Kralova *et al*. 2014; Kubat *et al*. 2014) and *Rumex acetosa* with XY1Y2 sex chromosomes (Steflova *et al*. 2013). Despite the apparently large size of the Y chromosome in *Coccinia grandis* attributed to extensive LTR retrotransposon accumulation (Souza *et al*. 2016), detailed investigations into specific TE clades and lineages within this species or in those with ZW sex determination systems remain scarce.

A close examination of the situation in the mentioned plants reveals that despite their ubiquity in the genome, most TE clades show one of two patterns on sex chromosomes: (i) some TE clades are accumulated on the Y chromosome while being less abundant on X than on autosomes, and (ii) others are almost absent on the Y chromosome while being more abundant on X than on autosomes (Fig. 1d). In addition, a bioinformatics study of flow‐sorted *R. acetosa* chromosomes showed that although the overall percentages of Ty3/Gypsy and Ty1/Copia superfamilies on the sex chromosomes are similar, the X chromosome is actually populated by different clades of LTR retrotransposons than the Y chromosome (Jesionek *et al*. 2021). All in all, distribution of TE clades on sex chromosomes seems to follow the prediction of the model proposed by Hobza *et al*. (2017), which assumes that TEs jump preferentially in either the male or female parental line, i.e. sex‐specifically. We discuss the potential causes of specific TE distribution on the sex chromosomes below.

## PROCESSES DETERMINING DISTRIBUTION OF TRANSPOSABLE ELEMENTS

Older studies identified two main mechanisms standing behind uneven chromosomal distribution of TEs: (i) selection processes retaining elements in a discrete genomic niches and removing them from other genomic parts (Fig. 2) and/or (ii) integration site preference, or targeting of elements into the specific sites in the genome (Bourque *et al*. 2018 and references within, Fig. 2).

Now, as our knowledge has increased, the situation is more complex and we are able to identify two “pre‐insertional” and two “post‐insertional” processes that result in various patterns of chromosomal localization of transposable elements: “Pre‐insertional” processes include (i) targeting into specific loci and (ii) sex‐specific transposition, while “post‐insertional” processes include (i) selection and (ii) ectopic recombination.

### The role of purifying selection in transposable element removal

The majority of TEs exhibit no specific insertion preferences in terms of primary sequence or secondary structure, resulting in their widespread presence across genomes. However, some of these originally randomly dispersed elements show uneven intrachromosomal distribution after some evolutionary time and gradually occupy specific niches. It is a result of purifying selection that removes elements from various genomic sites but retains these elements only in specific genomic regions, e.g. in subtelomeres (Fig. 2).

It is challenging to determine whether the uneven distribution of transposable elements (TEs) is due to targeting specific chromosomal regions or purifying selection. There is evidence supporting the role of purifying selection in determination of TE distribution. For example, in *Arabidopsis lyrata*, smaller populations accumulate transposable elements at higher frequencies due to increased genetic drift and reduced purifying selection (Lockton *et al*. 2008). Furthermore, in *Brachypodium distachyon*, recent population bottlenecks and demographic changes contribute to the loss of retrotransposons across genetic clusters (Stritt *et al*. 2018).

**Fig. 3:**
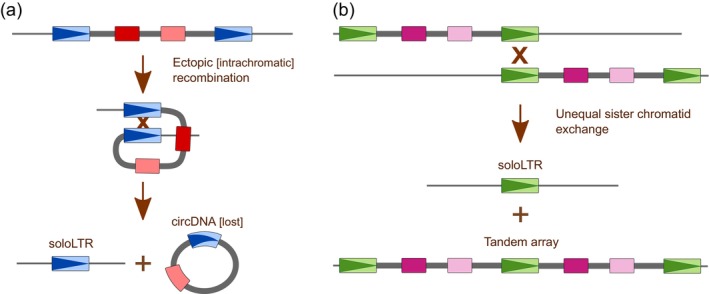
Removal of LTR retrotransposons by ectopic recombination. (a) Intrachromosomal ectopic recombination leaving behind a soloLTR and circular molecules in TE copies located on the same chromatid. (b) Intermolecular ectopic recombination of copies in different chromosomes resulting in soloLTRs and tandemly arranged TEs.

The mechanisms of element removal from the genome are represented by ectopic recombination (Fig. 3) removing either parts or the whole elements, unequal crossing‐over, and small or large DNA deletion. Such removal mechanisms are weaker in some regions, like e.g. subtelomeres in case of the *Retand* element of *Silene latifolia*, keeping the element there (Kejnovsky *et al*. 2006).

### Targeting of transposable elements into other elements (nesting): The role of the integrase protein and insertion into specific DNA motifs

Some TEs exhibit a certain level of integration site preference, from very specific target sites to non‐random but more dispersed genomic biases (Fig. 2). Short DNA motifs, epigenetic marks and nuclear proteins have been associated with such integration site preferences. The integrase of certain Ty3/Gypsy retrotransposons contains a chromodomain that can bind to repressive histone marks and aid insertion into heterochromatin (Gao *et al*. 2008).

Is the choice of TE target site biologically significant? The specificity of integration has important consequences for both the transposable element and the host cell (Craigie 1992). While insertions of TEs into active genes could be detrimental for the host organism, insertions into other TEs do not affect organism fitness. TEs are often nested into other TEs (Jedlicka *et al*. 2019). During this nesting, plant LTR retrotransposons end up in specific regions of pre‐existing transposable elements, namely the 3′UTR regions of other LTR retrotransposons while coding and regulatory regions (like LTRs) remain avoided (Jedlicka *et al*. 2019). The insertion bias is often affected by the presence of specific DNA motifs or by epigenetic marks recognized by the integrase enzyme coded by LTR retrotransposons.

Integrase, a specialized DNA recombination enzyme, is a key determinant of insertion specificity or site preference in LTR retrotransposons. LTR retrotransposons probably evolved from non‐LTR retrotransposons by acquisition of an integrase, possibly from a DNA transposon as the catalytic domain of integrases and transposases are related (Finnegan 2012). Retroviruses probably evolved from LTR retrotransposons in the Palaeozoic Era by incorporating envelope genes from other viruses (Aiewsakun & Katzourakis 2017; Hayward 2017). Bellow, we will describe integration mechanisms in DNA transposons, retroviruses and LTR retrotransposons, and non‐LTR retrotransposons:

- First, integrases of LTR retrotransposons evolved from a common ancestor as did the transposases of DNA transposons and recombinases (Capy *et al*. 1996). Both transposases and integrases, are the proteins that mediate transposition reactions, i.e. the recombination reaction in which discrete DNA segments move between nonhomologous sites. While mostly inherited vertically, they also may have spread between species by horizontal gene transfer. The analysis of 69 plant genomes and over 88,000 TE showed that LTR‐retrotransposon *Copia* clades sharing outstanding similarity between distant species were likely involved in horizontal gene transfer mechanisms, more frequent than initially estimated (Orozco‐Arias *et al*. 2023). DNA transposons possess the terminal inverted regions (TIR) that readily form complex secondary structures. Transposase binds both ends of DNA transposons and a DNA loop is formed during insertion into a new genomic site. Similar mode of action shows the integrase of LTR retrotransposons which binds the ends of retrotransposon cDNA.
- Second, retroviral integrase proteins have been reported to bind to numerous cellular proteins (Turlure *et al*. 2004) and some of these, like LEDGF/p75, have been confirmed to play an important role in integration. Thus interactions of integrase with cellular proteins have a clear biological relevance and help targeting the elements into specific genome sites. The integrase of chromoviruses possesses a chromodomain that assists (i) their targeting into centromeres (Kordis 2005) and (ii) interaction with the centromeric CENH3 protein which presumably determines their distribution (Sultana *et al*. 2017). Chromodomain can recognize not only specific DNA motifs but also specific epigenetic marks such as DNA methylation, histone acetylation, or changed DNA conformation (Gao *et al*. 2008). Moreover, recently the histone variant H2A.Z was determined to function as a guide for integration of certain Ty1/copia LTR retrotransposons in Arabidopsis (Roquis *et al*. 2021).
- Finally, in non‐LTR retrotransposons, the insertion site is selected by an endonuclease encoded by the retrotransposon followed by target‐primed reverse transcription (TPRT). Alternatively, reverse transcriptase starts the synthesis of cDNA at the single‐strand breaks caused by various physical and chemical agents (Cost *et al*. 2002).

A common phenomenon displayed by TEs is their nesting, i.e. insertion of one element into another, resulting among other things in the minimization of their potentially harmful effects on the host. The nested plant LTR retrotransposons showed preference for palindromes (Jedlicka *et al*. 2019) indicating a potential role of DNA secondary structure in TE insertion. Moreover, various plant and human transposable elements are often surrounded by microsatellite sequences that indicate the targeting of transposable elements into microsatellites which thereby serve as “landing pads” or “runways” for insertions (Kejnovsky *et al*. 2013). Again, this preference was strongest in microsatellites readily adopting unusual DNA conformation such as right‐handed A‐DNA or left‐handed Z‐DNA, clearly indicating the role of DNA conformation in the process of insertion (Kejnovsky *et al*. 2013). DNA with changed conformation is often nucleosome‐free and therefore is a better target for landing of some transposable elements (Gangadharan *et al*. 2010).

In addition, the chromosomal distribution of DNA transposons is shaped by “local hopping” defined as a phenomenon of chromosomal transposition in which transposons have a preference for landing into *cis*‐linked sites in the vicinity of the donor locus. “Local hopping” seems to be a shared feature of ‘cut and paste’ DNA transposons. For example, the *P*‐element transposon of *Drosophila* prefers to insert within ~100 kb of the donor site at a rate ~ 50‐fold higher than in regions outside that interval (Tower *et al*. 1993). The local hopping feature not only differs between different transposons, but a given transposon may show great variations in local hops in different hosts, and in different donor loci even in the same host. The frequency of local hopping greatly varies in *Arabidopsis* and tobacco, depending on the chromosomal location of the donor site (Dooner *et al*. 1991; Bancroft & Dean 1993).

### The role of sex‐specific jumping and the effect of DNA recombination on transposable element distribution

**Fig. 4:**
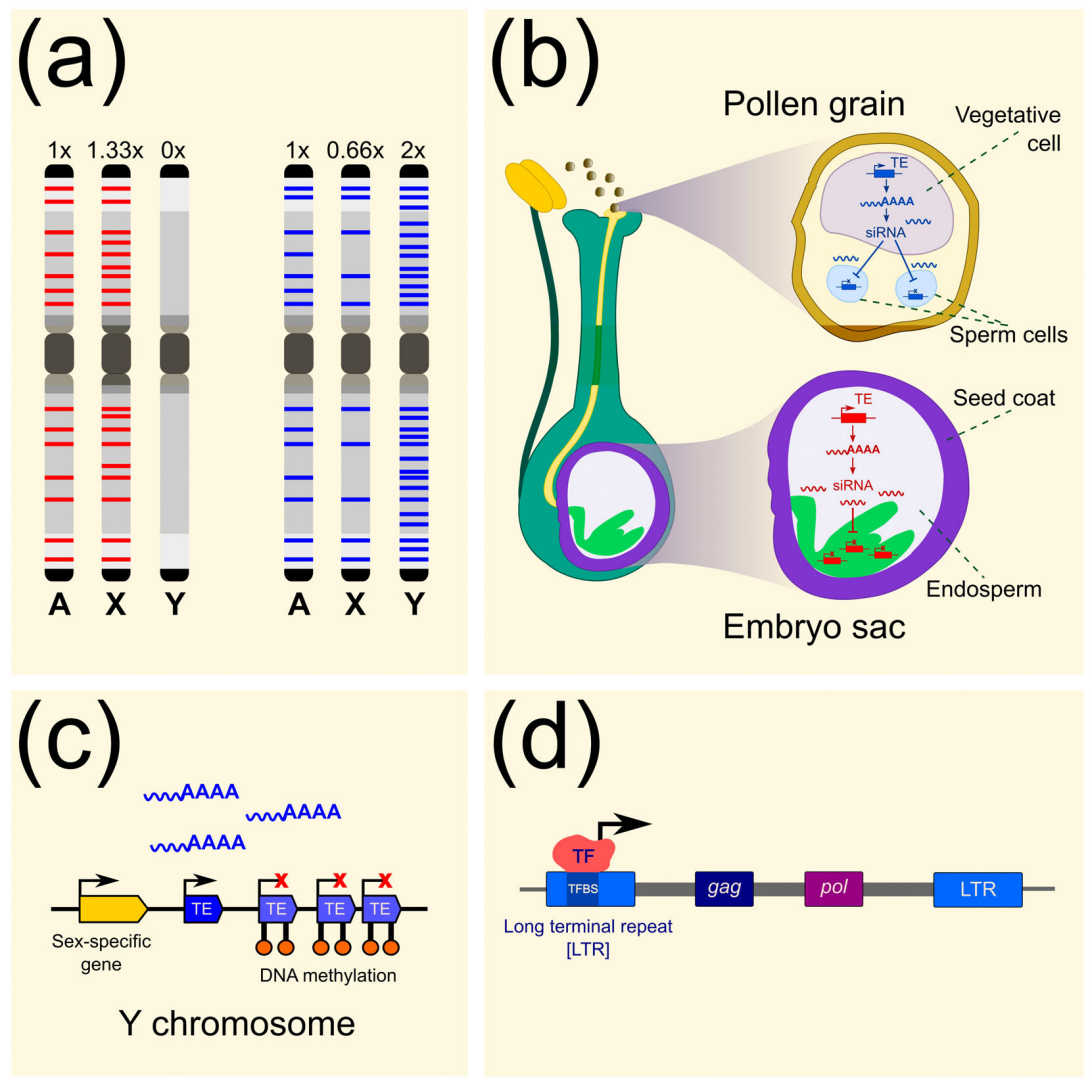
Possible mechanisms of sex‐specific retrotransposon jumping. (a) Simplified representation of TE distribution on heteromorphic non‐recombining sex chromosomes when a single TE lineage is shown in isolation. Left—insertion density of a TE lineage that is active maternally (red strips). Right—insertion density of a paternally active TE lineage (blue strips; adapted from Hobza *et al*. 2017). (b) TE lineages may not be silenced equally by epigenetic mechanisms. The figure shows a general scheme of epigenetic defence against TEs reactivated by epigenetic genome reprogramming in plant germlines and gametophytes. Vegetative cell of pollen grain with relaxed epigenetic silencing is a source of TE transcripts that are processed into siRNA molecules. The siRNAs move to sperm cells and ensure TE silencing. Similarly in female gametophyte, siRNAs are produced in the central cell of embryo sac, endosperm and seed coats. After relocalization into the egg cell and embryo, siRNAs ensure TE silencing (adapted from Law & Jacobsen 2010). (c) Colocalization of a TE master copy with sex‐biased gene can trigger sex‐specific bursts of activity. (d) Spatiotemporal regulation of TE transcription by transcription factors (TFs) may lead to either paternal or maternal proliferation. The diverse TE lineages contain binding sites for tissue‐specific TFs in their LTRs (Horvath *et al*. 2024). Depending on the identity of the TFs, TEs may be transcriptionally activated only transiently in certain cell types, eg. male or female sexual organs. The new genomic copies of TEs are then passed to the progeny via gametes. Different lineages of TEs are likely to preferentially use either male or female organs for their proliferation.

Sex chromosomes represent sex‐specific chromatin, regions of the genome that spend different evolutionary time in male and female individuals. The Y chromosome spends 100% of its evolutionary time in males. The X chromosome spends 1/3 of its evolutionary time in males and the remaining 2/3 in females. Only the autosomes are found equally in both sexes. Therefore, the accumulation or absence of TEs on sex chromosomes may reflect long‐term sex‐specific processes regulating transposon jumping (Fig. 4).

Figure 4a shows idealized insertion densities of either maternally or paternally jumping transposons. Actual chromosomal distributions may differ due to the peculiar nature of non‐recombining heteromorphic sex chromosomes such as the absence of meiotic recombination or the higher probability of DNA elimination and rearrangements due to unequal and illegitimate recombination (Fu *et al*. 2001; Tian *et al*. 2009), and the different selection pressures compared to autosomes. Although the general mechanism underlying sex‐specific transposition of TEs is not yet known, at least three potentially involved factors can be identified:

- First, epigenetic defence may differ in the male and female germline or the sporophytic cells that nourish it (Fig. 4b). This epigenetic defence is a response to reactivation of TEs following epigenetic reprogramming of the genome (Law & Jacobsen 2010; Kawashima & Berger 2014). The mechanism for reprogramming the paternal epigenome is known to differ from the maternal epigenome (Borg *et al*. 2020), providing an opportunity for divergence in the epigenetic regulation of individual TE clades. However, other factors may also play a role. For example, EVD elements in *A. thaliana* proliferate only paternally, whereas they are epigenetically silenced in the maternal lineage probably due to sporophytic epigenetic factors influencing female gametophyte (Reinders *et al*. 2013). Conversely, proliferation of TEs through the maternal but not the paternal lineage may be due to additional epigenetic defences specific to the male gametophyte, such as tRNA‐derived siRNAs (Martinez *et al*. 2017; Schorn *et al*. 2017) and miRNAs triggered siRNAs (Creasey *et al*. 2014) targeting certain TE families.
- Second, colocalization of TEs with sex‐biased genes can trigger sex‐specific bursts of activity (Fig. 4c). Because TEs have multiple negative effects on genes, they are either rapidly purged from the vicinity of active genes by purifying selection (Quadrana *et al*. 2016), or epigenetically silenced (Sigman & Slotkin 2016). Since the borders between TEs and genes are maintained by perpetual de‐novo DNA methylation of the TE ends and counteracting demethylation from the genic region, it is thought that epigenetic silencing can spread from TE insertion to a gene and compromise its expression (Bucher *et al*. 2012; Gent *et al*. 2013, 2014; West *et al*. 2014; Li *et al*. 2015; Vergara & Gutierrez 2017; Choi & Lee 2020). If the gene is essential, epigenetic conflict tips the balance towards that gene and the locus remains euchromatic, leading to TE transcriptional activity. If the gene is both essential and sex‐biased, TE transcription will be sex‐specific. Although this positional effect has not yet been seen in plants, a similar phenomenon has been described in fruit flies. Male fruit flies show higher embryonic expression of TEs located in the vicinity of essential genes on the Y chromosome (Wei *et al*. 2020). As a result, many TE lineages are accumulated on the Y chromosome in several Drosophila species (Nguyen *et al*. 2022).
- A third potential reason is spatiotemporal activity of TEs. When and where TEs produce new genomic insertions that are transmitted to subsequent generations remains a largely unresolved question. In somatic cells, TEs tend to be heterochromatinized and as such exhibit low transcript levels. Higher transcript levels are observed in shoot apical meristems (Ohtsu *et al*. 2007; Vicient 2010), developing germline and gametophyte, which is traditionally explained by epigenetic genome reprogramming, i.e. extensive removal of epigenetic silencing marks to restore totipotency of the fertilized egg (Gehring *et al*. 2009; Hsieh *et al*. 2009; Calarco *et al*. 2012; Calarco & Martienssen 2012; Gehring 2013). Haploid life stage characterized by relaxation of epigenetic regulation is therefore considered a prime candidate for the transgenerational spread of TEs. However, in light of the growing evidence that TEs contain transcription factor binding sites (TFBS) (Qiu & Köhler 2020), it is becoming increasingly apparent that TEs, like genes, require the binding of specific transcription factors (TFs) for their activation (Fig. 4d). Well‐supported is the activation of TEs by stress (Naito *et al*. 2009, 2014; Zeller *et al*. 2009; Ito *et al*. 2011; Zervudacki *et al*. 2018), which is facilitated by binding sites for stress‐related TFs (Cavrak *et al*. 2014; Pietzenuk *et al*. 2016; Deneweth *et al*. 2022). In *Brassica* species, many TEs contain TFBS for E2F, a family of TFs with various functions in cell proliferation and differentiation (Hénaff *et al*. 2014). Moreover, TEs were shown to rewire the regulatory network modulating flower development through transpositional spreading of MADS‐box TFs (Muiño *et al*. 2016; Baud *et al*. 2022), and contributed to evolution of flowering regulation responding to external factors (Quadrana *et al*. 2016; Quadrana 2020). Thus, it appears that transcriptional activation in meristems and reproductive organs is not solely a consequence of epigenetic relaxation, but is also a consequence of the binding of TFs regulating cell differentiation and floral development. This is further supported by a recent study examining the structure of LTR sequences and finding that LTRs are characterized by the presence of stress‐related TFBSs, TFBSs associated with expression in developing reproductive organs, and controlled DNA demethylation (Horvath *et al*. 2024). Thus, although it is not yet a well‐confirmed hypothesis, TEs, especially LTR retrotransposons, have probably adopted a strategy that allows them to activate only during reproduction, and ensures the transmission of new insertions to progeny without threatening the host with excessive insertional mutagenesis. It is because gametophytes represent a stage at which either male or female individuals (or both) are present in high numbers in many plants, allowing for potentially harmful TE jumping and the resulting selection process to take place without negative, and possibly even with positive (in terms of fitness) consequences for the population as a whole. In a similar fashion, meristems represent a system of pluripotent cells where potentially harmful TE jumping in individual cells can be tolerated. Individual lineages of TEs likely differ in the specific configuration of TFBS, which is manifested in transient activation in either male or female reproductive organs and diverse stages of their development. A side effect may be an uneven density of insertions on the sex chromosomes, either accumulation on the Y or on the X, for TEs activated in either male or female reproductive organs, respectively.

The sex‐specific distribution of TEs provides an opportunity to determine how and when TEs reproduce themselves during the plant life cycle. To answer this puzzle and decide between three mentioned scenarios, several questions still need to be addressed: (i) Do diverse TEs spread either maternally or paternally, as predicted by the model proposed by Hobza *et al*. (2017)? (ii) Is parent‐specific proliferation a conserved trait of the particular TE clades across separate populations of a species and in closely related species? (iii) Can the association of floral organ‐specific TFs with TEs be validated and linked to transgenerational TE proliferation?

Taken together, after the TE insertion into the sex‐chromosomes by any of the above mentioned scenarios, various recombination and selection processes acting on the Y and X chromosomes influence the final TE distribution pattern. However, the previous hypothesis (Charlesworth 1991) expecting strong accumulation of any TEs on any non‐recombining Y chromosome seems to overestimate the effect of recombination restriction on TEs accumulation. Although this accumulation was observed in the Y chromosome, it does not explain why many abundant TEs are absent on the Y chromosome in *Rumex acetosa* and *Silene latifolia* (Cermak *et al*. 2008; Jesionek *et al*. 2021; Moraga *et al*. 2023).

## FUNCTIONAL CONSEQUENCES OF CHROMOSOMAL LOCALIZATION OF TE

When considering the functional consequences of TE distribution, it is important to move beyond the classical gene‐centered evolutionary thinking and use the genome‐centric concept (Heng 2009). In this view, the genome is a dynamic system where interactions of many sequence elements—not only genes—can contribute to both cell functioning and genome evolution. Chromosomal localization of TEs in relation to genes and different types of chromatin (heterochromatin vs euchromatin) can both influence activity of individual genes and silence larger genomic regions. The gathering of TEs in specific chromosomal niches and the physical interactions between TEs in 3D interphase nucleus are important for the final genome activity and for the cellular molecular concert. Understanding these interactions not only provides insights into gene regulation but also highlights the complex interplay between TEs and other genomic elements in shaping evolutionary trajectories. Movement of various DNA and RNA molecules plays a role in these processes and contributes to the genome dynamism (Kejnovsky & Jedlicka 2022).

Like genes, transposable elements (TEs) often contain regulatory elements such as promoters, enabling them to influence not only their own transcription but also the expression of downstream genes. Furthermore, the silencing of TEs through epigenetic mechanisms can lead to the conversion of TE‐rich regions into heterochromatin. This process can regulate gene expression by spreading silencing marks to neighbouring genes that would otherwise be expressed (Choi & Lee 2020).

Transposable elements often carry motifs contributing to the regulation of cellular processes. Long terminal repeats of LTR retrotransposons, for example, contain transcription factor binding sites (TFBS) thus contributing to the formation of a wired network (Qiu & Köhler 2020; Zhang *et al*. 2021). Moreover, TEs can provide other regulatory DNA motifs, e.g guanine quadruplexes, and spread them in the genome (Kejnovsky & Lexa 2014). By regulating the abundance of quadruplexes in the genome, TEs can contribute to the regulation of replication, recombination and chromatin remodelling, processes in which G4s play an important role (Kejnovsky & Lexa 2014). Other sequence modules provided by TEs may be at play here as well, such as small RNAs (Feschotte 2008) and replication origins (Wyrick *et al*. 2001).

The high number of similar copies of TEs can be viewed as paralogs forming “families” (analogous to gene families in the gene world) that are dispersed in the genome and can build functional networks on transcriptional level via parallel interactions with transposase‐derived transcription factors (Feschotte 2008) or on the genome level via mutual recombination during the process of reverse transcription (Drost & Sanchez 2019). Such functional networks based on the multicopy character of TEs are advantageous especially when transposase or some other DNA‐binding TE component is fused with another cellular gene or protein domain that enables the targeting of many genomic sites simultaneously.

Perhaps because of their promiscuous nature, TEs and their components also seem to be especially prone to adaptive modifications to functions outside the realm of simple TE reproduction. This evolutionary process is called domestication or exaptation (also co‐option) of TEs (Joly‐Lopez & Bureau 2018). A survey of existing literature shows these three terms used interchangeably (Joly‐Lopez & Bureau 2018; Almeida *et al*. 2022 and references therein). On the other hand, Schrader & Schmitz (2019) distinguish domestication (or co‐option) from exaptation, reserving the latter for cases where the TE or its component only become a part of a larger functional unit, typically a protein. Capy (2021) proposes to use the term “exaptation” only for cases where domestication leads to maintenance of new functions and sometimes new genes, which significantly increase host fitness and become fixed in the population or even several species.

Many cases of domestication/exaptation involving TEs have been studied in plants. Schrader & Schmitz (2019) provide an exhausting list in their work. Examples of domestication/exaptation are often found in cultivated crops as a result of breeding, or in wild populations undergoing adaptation. Some of these represent a simple mutational event. For example, Goettel et al. (2022) identified a TE insertion responsible for truncating a domain of the POWR1 gene in soybean affecting seed oil content and yield. On the other side of the spectrum is the well‐known *SETMAR* gene, originated by the fusion of transposase with histone‐methyltransferase that resulted in the changes in overall methylation status of histones. Similarly, the insertions of the MITE family, Monkey King, are utilized in Brassicaceae genes for gene expression (Dai *et al*. 2015). Both Velanis *et al*. (2020) and Liang *et al*. (2015) describe another transposase domestication in the evolution of PRC2 from the polycomb repressive complex in Arabidopsis. Niu *et al*. (2019) describe modulation of flowering time variation in *Capsella rubella* as a result of TE activity in the population, a survival‐linked phenotype that can easily mediate long‐term evolutionary cross‐talk. Modzelewski *et al*. (2022) review similar cases and their underlying mechanisms known in the animal kingdom.

Among many cases found so far, a remarkable example of transposable element domestication is represented by the telomeric retrotransposons of various eukaryotes that protect chromosome ends instead of telomeric satellites common in other species (Jedlička *et al*. 2023). Although not found in plants yet, these retrotransposons carry abundant strand‐biased G4 motifs that support the role of G4s and recruitment of these retrotransposons for chromosome end protection, similarly as G4 formation in traditional telomeres (Jedlička *et al*. 2023).

Moreover, sequentially similar repeats, including TEs, are more often in contact than different repeats (Lexa *et al*. 2022). We can speculate that such interactions in 3D interphase nucleus can help certain molecular processes, especially those that are positional in their nature, such as regulation (cis or trans) or recombination. These also include gene conversion homogenizing similar repeats that can be later removed by ectopic recombination (Fig. 3). This way TEs participate in genome expansion (amplification of TEs) as well as contraction (removal of elements by ectopic recombination) shaping the genome size.

## CONCLUSIONS AND PERSPECTIVE

In this review, we described not only the variability of chromosomal niches selected by various TEs for their survival mostly in plant genomes but also the processes standing behind TE localization. It is now evident that the chromosomal distribution of TEs results from an interplay between the inherent properties of various TE families and their interactions with the broader genomic landscape, including genes. Both groups, TEs and genes, affect each other, e.g. by epigenetic cross‐talk, or providing material for fragments or entire promoters from which members of the other group are transcribed.

It is now becoming increasingly clear that cell function and genome evolution are also correlated to 3D genome folding. Transposable elements as “living elements” within a complex genomic environment are intrinsically linked to 3D organization (Bousios *et al*. 2020). All above‐mentioned processes and modes of spreading make TEs a separate class of DNA sequences bringing an extra set of functionalities that allow the genome and the cell to be relatively quickly shaped by various evolutionary processes. TEs represent a versatile genomic tool that can sensitively respond to the (i) short‐term environmental changes by changing the reversible epigenetic layer of information as well as respond to the (ii) long‐term environmental changes by heritable genetic changes allowed by their mobility. Moreover, the networking effect of TEs ensures the functioning of the genome and the cell in a concert as a fine‐tuned functional unit.

## AUTHOR CONTRIBUTIONS

EK conceived a basic idea of the review and designed its main structure. EK, PJ, ML and ZK wrote the manuscript. PJ prepared the Figures. All authors read and approved the manuscript.

## FUNDING INFORMATION

This work was supported by the grants 21–00580S and 24‐11400S from the Czech Science Foundation awarded to E.K. and the grant 22‐00364S to Z.K. The work was further supported from the project TowArds Next GENeration Crops, reg. no. CZ.02.01.01/00/22_008/0004581, within the ERDF Programme Johanes Amos Comenius.

## CONFLICT OF INTEREST

On behalf of all authors, the corresponding author states that there is no conflict of interest.

## Data Availability

Raw image data is freely available in the Zenodo data repository https://doi.org/10.5281/zenodo.15480284, (Kejnovský *et al.*, 2025).
